# Angiogenesis Inhibitors in Small Cell Lung Cancer

**DOI:** 10.3389/fonc.2021.655316

**Published:** 2021-05-28

**Authors:** Agnese Montanino, Anna Manzo, Guido Carillio, Giuliano Palumbo, Giovanna Esposito, Vincenzo Sforza, Raffaele Costanzo, Claudia Sandomenico, Gerardo Botti, Maria C. Piccirillo, Priscilla Cascetta, Giacomo Pascarella, Carmine La Manna, Nicola Normanno, Alessandro Morabito

**Affiliations:** ^1^ Thoracic Department, Istituto Nazionale Tumori, IRCCS “Fondazione G.Pascale”, Naples, Italy; ^2^ Department of Oncology and Hematology, Azienda Ospedaliera Pugliese-Ciaccio, Catanzaro, Italy; ^3^ Scientific Directorate, Istituto Nazionale Tumori, “Fondazione G. Pascale” - IRCCS, Naples, Italy; ^4^ Scientific Department, Istituto Nazionale Tumori, “Fondazione G.Pascale” – IRCCS, Naples, Italy; ^5^ Oncology Department, University Federico II, Naples, Italy

**Keywords:** small cell lung cancer, angiogenesis, vascular endothelial growth factor, bevacizumab, anlotinib

## Abstract

Inhibition of angiogenesis has been demonstrated to be an efficacious strategy in treating several tumors. Vascular endothelial growth factor (VEGF) is the most important protein with proangiogenic functions and it is overexpressed in small cell lung cancer (SCLC). Bevacizumab, a monoclonal antibody directed against VEGF, showed a promising activity in combination with etoposide and cisplatin as first-line treatment of patients with extended stage (ES)-SCLC and two randomized studies confirmed that bevacizumab improved PFS, but failed to prolong OS. Instead, disappointing results have been observed with endostar, sunitinib, sorafenib, vandetanib, and thalidomide in combination with chemotherapy in the first-line setting, with sunitinib in the maintenance setting, with sunitinib, cediranib and nintedanib as single agents or ziv-aflibercept in combination with topotecan in second-line setting. Only anlotinib improved OS and PFS as third-line therapy in Chinese patients with SCLC, and it was approved with this indication in China. Future challenges are the evaluation of the role of angiogenesis inhibitors in combination with immune- checkpoint inhibitors and chemotherapy in SCLC patients and the identification of predictive biomarkers of response to both agents.

## Introduction

Lung cancer is the most frequent cause of tumor death worldwide. Small cell lung cancer (SCLC) accounts for 10% to 15% of all lung cancers and tobacco is a universally recognized inducing factor (1). In one third of cases SCLC presents as a limited stage disease confined in a hemi-thorax. In about 70% of cases, SCLC is diagnosed in extensive stage, when the disease has already spread elsewhere. Strategy for the treatment of SCLC has made no relevant progress in the last four decades and the first-line standard therapy remained a combination of platinum compounds and etoposide until few years ago (2). Despite a high response rate to first-line treatment, the recurrence is generally unavoidable and the progression of disease becomes then rapid and invariably lethal. Ultimately, novel immune check point inhibitors revealed efficacy in prolonging overall survival, and they were included in the first-line treatment in combination with chemotherapy (3). Angiogenesis is a crucial pathway exploited by many tumors to escape from the physiologic control and immune surveillance. Deregulated mechanisms of angiogenesis confer to the tumor the capability of growing indefinitely. Vascular endothelial growth factor (VEGF) is the most important protein with proangiogenic functions (4). VEGF family includes VEGF-A, which stimulates angiogenesis and vascular permeability by binding to its receptors, VEGFR-1 (Flt-1) and VEGFR-2 (KDR/Flk1). VEGF-C and VEGF-D bind to another receptor, VEGFR-3 (Flt-4), regulating lymphangiogenesis. Inhibition of VEGF or VEGFR has been demonstrated an efficacy strategy in treating several tumors, including colon, stomach, breast, ovary, thyroid, kidney, liver, and non-small-cell lung cancers. SCLC proliferation has been demonstrated strictly dependent from microvessels formation (5). Moreover, VEGF is over-expressed in SCLC, and it is associated to poor prognosis. Therefore, inhibition of angiogenesis could represent a promising strategy of treatment for SCLC. In this review, we will summarize the main results obtained by targeting angiogenesis pathway with a monoclonal antibody anti VEGF (bevacizumab) or with small-molecule tyrosine kinase inhibitors of VEGFR in SCLC.

## Bevacizumab in SCLC

Bevacizumab is a humanized monoclonal antibody directed against VEGF that binds to all circulating soluble VEGF-A isoforms, preventing the interaction of VEGF-A with VEGFR and thereby inhibiting the activation of VEGF signaling pathways that promote neovascularization (6). Bevacizumab blocks vessels growth, induces regression of newly formed vessels, and normalizes the vasculature, facilitating the delivery of cytotoxic chemotherapy, but it has also direct effects on tumor cells (7). It was the first anti-angiogenetic treatment approved by the Food and Drug administration (FDA) in combination with chemotherapy for metastatic colon cancer, and then for different other tumors including kidney, ovarian, lung cancer and recurring glioblastoma multiforme (8, 9). Bevacizumab has also been evaluated in SCLC with contrasting results, both in limited and extended disease (**Table 1**).

**Table 1:** Bevacizumab in ES-SCLC.

Author	Regimen	Patients	ORR (%)	Median TTP/PFS (months)	Median OS (months)	1-year OS (%)	Grade 3-4 toxicity (%)
**Spigel DR et al., LUN90**	CaIB	51	84	9.1	12.1	51	Thrombocytopenia (53), fatigue (31), dehydration (26), diarrhea (21), hyperglycemia (21), pain (21)
**Horn L et al., E3501**	CEB	63	63.5	4.7	10.9	38.1	Neutropenia (57.8), thrombocytopenia (14.1), fatigue (14.1)
**Ready NE et al., CALGB30306**	CIB	72	75	7.0	11.6	43.8	Neutropenia (25), diarrhea (16), dehydration (12), thrombocytopenia (10), fatigue (10), nausea (10)
**Spigel DA et al., SALUTE**	Ca/CEB vs Ca/CEP	102	58 vs 48	5.5 vs 4.4	9.4 vs 10.9	–	Neutropenia (35), pneumonia (5.9), dyspnea (3.9), thrombocytopenia (4), hypertension (5.9)
**Pujol JL et al., IFCT-0802**	CT vs CT + B	147	89.2 vs 91.9, p=1.00	5.5 vs 5.3, p=0.82	13.3 vs 11.1, p=0.80	–	Hypertension (40), thrombosis (11)
**Tiseo M et al., FARM6PMFJM**	CEB vs CEP	204	58.4 vs 55.3, p=0.657	6.7 vs 5.7, p=0.03	9.8 vs 8.9, p=0.11	37 vs 25	Neutropenia (46.3), fatigue (8.4), hypertension (6.3), thrombosis (5.3)

Ca, carboplatin; C, cisplatin; I, irinotecan; E, etoposide; B, bevacizumab; P, placebo; CT, chemotherapy chosen by each center, cisplatin etoposide or cisplatin-cyclophosphamide-epidoxorubicin-etoposide.

In patients with LS-SCLC, a multicenter phase II trial of the Minnie Pearl Cancer Research Network evaluated the activity of bevacizumab as maintenance therapy following treatment with carboplatin, irinotecan and concurrent radiation (10). From August 2003 to October 2004, 57 patients were enrolled in this study and received four cycles of carboplatin AUC 5 on day 1, irinotecan 50 mg/m2 on days 1 and 8 and from the third cycle radiotherapy 1.8 Gy daily for a total of 61.2 Gy. After four cycles, if there was no progressive disease, patients received bevacizumab 10 mg/kg every 14 days for ten cycles. Median overall survival (OS) was 15 months; 1- and 2-year progression-free survival (PFS) rates were 63% and 54%, respectively, response rate was 80%. Two deaths were observed due to respiratory failure; 9% of patients experienced G3-4 toxicity during bevacizumab therapy, including deep vein thrombosis and colon perforation. Therefore, this study did not support a further evaluation of bevacizumab as maintenance therapy in this setting of patients with LS-SCLC.

Several phase II studies have been conducted to date with bevacizumab in patients with ES-SCLC. The LUN90 trial was a phase II study that evaluated the activity of the combination of irinotecan, carboplatin, and bevacizumab in 51 patients with histologically confirmed ES-SCLC enrolled from February 2006 to March 2007 (11). All patients received carboplatin AUC4 intravenously on day 1 and irinotecan at a dose of 60 mg/m2 on days 1, 8, and 15, every 28 days for a maximum of six cycles. Bevacizumab (10 mg/kg) was administered on days 1 and 15 every 28 days. Six cycles of chemotherapy and bevacizumab were completed by 57% of patients. The ORR was 84%, with median response duration of 6.4 months. Median PFS was 9.13 months (95% CI, 7.36–9.46 months), and median OS was 12.1 months (95% CI, 9.6–13.5 months); 1- and 2-year overall survival rates were 51% and 14%, respectively. The treatment was well tolerated without grade 3 or 4 bleeding or stroke. However, two patients died due to infection, possibly related to treatment and another patient died for liver failure. The most frequent adverse events during combination were diarrhea and hematological toxicity. Other potential treatment-related toxicities were pulmonary embolism, acute renal failure and grade 4 hypertension occurring in one patient each. The subsequent Eastern Cooperative Oncology Group Study E3501 evaluated the activity of the combination of bevacizumab (15 mg/kg) with cisplatin (60 mg/m2) on day 1, etoposide (120 mg/m2) in ES-SCLC patients (12). Overall, 63 patients were included in the study: the response rate was 63.5%. Median PFS and OS were 4.7 and 10.9 months, respectively, that compared favorably with historical controls. The 6-month PFS was 30.2% and the 1-year OS was 38.1%. Patients with an ECOG performance status (PS) greater than 0 had an increased risk of progression or death compared with patients with PS of 0. Moreover, the relationship between baseline and changes in plasma VEGF, soluble cell adhesion molecules, and basic fibroblast growth factor and outcome was explored, but no association was observed. Bevacizumab associated toxicities included hypertension (7.8%), epistaxis (9.4%), grade 3 pulmonary hemorrhage in a patient and grade 3 abdominal hemorrhage in another patient. Two grade 4 bevacizumab associated adverse events were cardiac ischemia and thromboembolism. Two patients died, one due to multiorgan failure and one due to lung infection with grade 4 neutropenia. Another phase II study evaluated a different schedule of chemotherapy with cisplatin (30 mg/m2) and irinotecan (65 mg/m2) on days 1 and 8, in combination with bevacizumab (15 mg/kg) on day 1 every 21 days for six cycles (CALGB30306) in 72 patients with ES-SCLC (13). In this study, Bevacizumab was not continued after chemotherapy. The ORR was 75%: 5% of patients had a complete response and 70% a partial response. Median PFS was 7.0 months and median OS was 11.6 months. It was observed a significant association between the development of hypertension and improved survival after adjusting for age and performance status. In particular, OS was 10.7 months (95% CI, 8.4–12.9) for patients not experiencing hypertension, while it was 15.8 months (95% CI, 10.5–21.8 months) for patients experiencing ≥grade 1 hypertension. However, 1-year OS was 43.8%, less than the pre-specified value in the protocol of 57%: therefore, the study did not meet the primary objective of survival and this treatment regimen was considered not worthy for additional investigation. Moreover, three patients died during the study for pneumonitis, stroke, and heart failure. In 2011 Spigel et al. published the results of SALUTE trial, the first placebo-controlled, double blind, randomized multicenter phase II clinical trial to assess the efficacy and safety of bevacizumab added to doublet chemotherapy for treatment of ED-SCLC, with ECOG PS 0–2 (14). Patients were randomly assigned to receive bevacizumab plus chemotherapy or placebo plus chemotherapy, and they received four cycles of cisplatin 75 mg/m2 or carboplatin AUC5 on day 1, etoposide 100 mg/m2 on days 1 through 3 and bevacizumab at 15 mg/kg or placebo. After four 21-day cycles, patients received a maintenance therapy with bevacizumab or placebo: fifty-two patients were enrolled in bevacizumab arm and 50 patients in placebo arm. Median PFS was 4.4 months (95% CI, 4.2–4.9) in placebo arm and 5.5 months (95% CI, 4.5–6.7) in bevacizumab arm. An exploratory subgroup analysis showed a PFS benefit in patients who received bevacizumab plus carboplatin compared with patients treated with bevacizumab plus cisplatin or placebo plus either platinum type, although these results should be interpreted with caution because the relatively small sample sizes in the two cohorts. Moreover, the addition of bevacizumab to chemotherapy did not lead to an improvement in OS, which was 9.4 months versus 10.9 months in placebo and bevacizumab group respectively. Grade 3 to 5 adverse events (AEs) occurred more frequently in bevacizumab arm (75%) than in placebo arm (60%). There were four deaths in the study, two in bevacizumab group and two in placebo group, one of this death (a case of hemoptysis) was attributed to bevacizumab. The role of bevacizumab in SCLC patients who received two induction cycles of chemotherapy was explored by a French, randomized phase II to III study, the IFCT-0802 trial (15). Responder patients were randomized to receive four additional cycles of chemotherapy alone or in combination with bevacizumab, followed by single-agent bevacizumab until progression or unacceptable toxicity. The chemotherapy regimen was chosen by each center and it included cisplatin and etoposide or a four-drug regimen with cisplatin, cyclophosphamide, epidoxorubicin, and etoposide. From September 2009 to October 2011, 147 patients received two cycles of chemotherapy, 103 of these were responders, and 74 were randomized to receive chemotherapy alone or chemotherapy plus bevacizumab. There were no differences between the two groups in PFS or OS. Median PFS was 5.5 months (95% CI, 4.9%–6.0%) in chemotherapy alone versus 5.3 months (95% CI, 4.8%–5.8%) in chemotherapy plus bevacizumab group. Median OS, calculated from the date of randomization, was 13.3 months (95% CI, 9.8%–16.6%) versus 11.1 months (95% CI, 8.7%–14.0%) in the chemotherapy alone and chemotherapy plus bevacizumab arm, respectively. Therefore, the triplet with bevacizumab did not improve patient outcome compared to chemotherapy alone after induction chemotherapy.

Finally, a randomized controlled phase III trial was conducted by GOIRC-AIFA to definitively assess the efficacy of bevacizumab in combination with first-line cisplatin plus etoposide for the treatment of ED-SCLC (16). Two hundred five patients from 29 Italian centers were randomly assigned in the two arms and 204 patients were considered in the intention-to-treat analysis: 103 patients received cisplatin 25 mg/m2 on days 1 through 3, etoposide 100 mg/m2 on days 1 to 3 and bevacizumab 7.5 mg/kg on day 1 every 3 weeks for six cycles; 101 patients received the same chemotherapy regimen without bevacizumab. In case of cisplatin contraindications or toxicity the investigators could use carboplatin AUC5 on day 1. In experimental arm, bevacizumab as single agent was continued as maintenance therapy until progression or for a maximum of 18 cycles including the first six cycles. Prophylactic cranial irradiation (PCI) was allowed. No significant differences in hematological toxicity was observed between the two arms, while for non hematologic toxicity, grade 3 to 4 hypertension was more frequent in bevacizumab than control arm (6.3% versus 1.0%, p=0.057). The response rate was similar in the two arms (55.3% for chemotherapy alone versus 58.4% for experimental arm; HR, 1.13; 95% CI, 0.65–1.97; p = 0.657). Chemotherapy with bevacizumab led to a small, but statistically significant improvement in PFS (5.7 versus 6.7 months in the standard and experimental arm, respectively; HR, 0.72; 95% CI, 0.54–0.97; p = 0.030). However, no difference in OS was observed: median OS was 8.9 versus 9.8 months, and 1-year survival rate was 25% versus 37% (HR, 0.78; 95% CI, 0.58–1.06; p = 0.113) in chemotherapy alone group versus chemotherapy plus bevacizumab, respectively. The delivery of PCI was associated with a survival benefit (HR, 0.53; 95% CI, 0.29–0.98; p = 0.034). A subgroup analysis showed a statistically significant interaction for OS between treatment and sex: the addition of bevacizumab to chemotherapy led to a significant survival benefit in men and to a possible detrimental effect in women. Moreover, a significant effect on OS was observed with the maintenance treatment with bevacizumab (HR, 0.60; 95% CI, 0.40–0.91; p=0.011), generating the hypothesis that a sequential treatment with bevacizumab could be a better and safer strategy to deliver antiangiogenic drugs in SCLC.

## Other Angiogenesis Inhibitors in SCLC

Other antiangiogenic agents, including antibodies (ziv-aflibercept) and small molecules agents (rh-endostatin, vandetanib, sunitinib, sorafenib, cediranib, nintedanib, thalidomide, and anlotinib) have been tested in SCLC (**Table 2**).

**Table 2:** Other angiogenesis inhibitors in SCLC.

Author	Regimen	Setting	Patients	ORR (%)	Median TTP/PFS (months)	Median OS (months)	1-year OS (%)	Main toxicities
**Allen JW et al., 2014**	Aflibercept + topo vs topo	Pl-sensitive Pl-refractory	83	2 vs 0	1.8 vs 1.3	6 vs 4.6	–	Fatigue, gastrointestinal, bleeding, pulmonary
			106	2 vs 0	1.4 vs 1.4	4.6 vs 4.2	–	
**Zhou ZT et al., 2011**	CE + endostar	First line	33	69.7	5.0	11.5	38.1	Fatigue, nausea, diarrhea, anorexia, mucositis
**Lu S et al., 2018**	CBDCA-VP16 + endostar vs CBDCA-VP16	First line	140	75.4 vs 66.7	6.4 vs 5.9	12.1 vs 12.4	50.0 vs 54.6	Neutropenia, anemia, weakness, vomiting
**Arnold AM et al., 2007**	Vandetanib vs Placebo	Maintenance	107	–	2.7 vs 2.8	10.6 vs 11.9	–	Gastrointestinal, rash, QT prolongation
**Sanborn RE et al., 2016**	Pl +E + vandetanib vs Pl+E	First line	74	50 vs 65	5.62 vs 5.68	13.24 vs 9.23	–	Cardiac, hyperglycemia, hypertension
**Han JY et al, 2012**	Sunitinib	Second line	25	9%	1.4	5.6	21	Thrombocytopenia, asthenia, neutropenia
**Schneider BJ et al., 2011**	Sunitinib	Maintenance	16	–	6.2*	8.2*	–	Thrombocytopenia, fatigue, muscle weakness, hypothyroidism
**Spigel DR et al., 2012**	Sunitinib	Maintenance	17	–	7.6*		54	Thrombocytopenia, anemia, vomiting, fatigue, pain, dehydration
**Ready NE et al., 2015**	Sunitinib vs placebo	Maintenance	85	–	3.7 vs 2.1	9 vs 6.9	62.6 vs 43.9	Fatigue, neutropenia, thrombocytopenia
**Sharma N et al., 2014**	CE plus concurrent and sequential sorafenib	First line	18	47	–	7.4	25	Fatigue, anorexia, rash, diarrhea, neutropenia, weight loss, bleeding
**Ramalingam SS et al., 2012**	Cediranib	Second line	25	0	2	6	–	Fatigue, diarrhea, hypertension, proteinuria, elevated liver enzymes
**Han JY et al., 2016**	Nintedanib	Second line	24	5	1	9.8	–	Elevated liver enzymes, anemia, thrombocytopenia, neutropenia, anorexia, fatigue, diarrhea, vomiting
**Dowlati A et al., 2007**	Thalidomide	Maintenance	30	–	2.8	12.8*	51.7*	Neuropathy, constipation, fatigue, rash, dyspnea pulmonary embolism
**Lee SM et al., 2007**	CT+ thalidomide followed by thalidomide	First line and maintenance	25	68	8.3	10.1	42	Nausea, anorexia, drowsiness, rash
**Pujol JL et al., 2007**	PCDE + thalidomide vs PCDE + placebo	First line	119	87 vs 84	6.6 vs 6.4	11.7 vs 8.7	49 vs 30	Neutropenia, anemia, neuropathy, constipation
**Lee SM et al., 2009**	Ca + E + thalidomide vs Ca + E +placebo	First line	724	74 vs 72	7.6 vs 7.6	10.1 vs 10.5	37 vs 41	Thrombosis, rash, constipation, neuropathy
**Cheng Y et al., 2018**	Anlotinib vs placebo	Third line	120	71.6 vs 13.2^	4.3 vs 0.7	7.3 vs 4.9	–	Hypertension, anorexia, fatigue, elevation of liver enzymes, bleeding,
**Xu Y et al., 2019**	Apatinib	Third-fourth line	40	17.5%	3.0	5.8	–	Hypertension, hand-foot syndrome, increased GGT°
**Liu Y et al., 2020**	Apatinib	Third-fourth line	22	13.6%	5.4	10.0	–	Hypertension, proteinuria
**Fan Y et al., 2021**	Apatinib + camrelizumab	Second-line	47	34%	3.6	8.4		Hypertension, decreased platelet count, hand-foot syndrome

Topo, topotecan; Pl, platinum; E, etoposide; C, cisplatin; Ca, carboplatin; PCDE, etoposide, cisplatin, cyclophosphamide, epidoxorubicin.

* From the start of chemotherapy.

^ Disease control rate.

°Gamma-glutamyltransferase.

### Ziv-aflibercept

Ziv-aflibercept is a recombinant fusion protein that targets the vascular endothelial growth factor (VEGF) receptors that play a key role in tumor growth and metastasis. In a phase II trial, this drug was evaluated in pretreated SCLC patients in combination with weekly topotecan (17). Overall, 189 patients were randomized to receive weekly topotecan 4 mg/m2 intravenously (IV) with or without ziv-aflibercept 6 mg/kg IV every 21 days. Patients were stratified as platinum-sensitive or platinum-refractory. The 3-month PFS was improved by Ziv-aflibercept only in patients who had platinum-refractory SCLC (27% versus 10%, p=0.02), but not in patients with platinum-sensitive disease. Response rate was low, but disease control rate was slightly higher with combination therapy than with topotecan alone (37% versus 18%; p=0.05 in platinum-sensitive disease; 25% versus 15%, p=0.14 in patients with refractory disease). Moreover, no difference in overall survival was observed and Ziv-aflibercept increased grades 3 to 5 toxicity.

### Endostar

Endostatin, the 20-kD internal fragment of the carboxyterminus of collagen XVIII, was first identified in 1997 by Folkman et al. as a potent inhibitor of angiogenesis, blocking endothelial cell proliferation and migration, inducing endothelial cell apoptosis and cell cycle arrest (18). Endostar, a novel recombinant human (rh)endostatin purified in *E. coli*, was evaluated in a single-arm phase II trial in combination with chemotherapy in patients with extensive stage (ED) SCLC (19). Thirty-three patients were treated with cisplatin 25 mg/m2 intravenously on days 1 to 3, etoposide 120 mg/m2 intravenously on days 1 to 3 and Endostar 15 mg by IV infusion on days 1 to 14 every 3 weeks. The median PFS (primary endpoint) was 5.0 months (95% CI, 4.2–5.6 months), and the 6-month PFS was 33.3%. Based on these results, the addition of Endostar to chemotherapy was then tested in a randomized phase II trial (20). One hundred forty patients with ES-SCLC were randomized to chemotherapy alone (cisplatin and etoposide) or rh-endostatin plus chemotherapy for four to six cycles, followed by single-agent rh-endostatin until progression or unacceptable toxicity. However, the trial failed to demonstrate the superiority of the combination of rh-endostatin plus chemotherapy. There was no difference between the two arms in the incidence of non-hematological or severe hematological toxicity.

### Vandetanib

Vandetanib (ZD6474) is a multitarget tyrosine kinase inhibitor targeting VEGFreceptor 2 (VEGFR-2), EGFR, VEGFR-3, and RET (21). This TKI was first evaluated in a double-blind randomized phase II trial as maintenance therapy in patients with SCLC who have responded to chemotherapy (22). A total of 107 patients were randomly assigned to vandetanib 300 mg/die or placebo arm. The trial failed to demonstrate the efficacy of vandetanib as maintenance treatment: median PFS was 2.7 and 2.8 months for vandetanib and placebo, respectively (HR, 1.01; 80% CI, 0.75–1.36; one-sided p = 0.51). Moreover, patients treated with vandetanib maintenance therapy had more adverse events, in particular asymptomatic corrected QT interval (QTC) prolongation. Another double blind, placebo-controlled phase II trial was conducted with vandetanib in combination with platinum and etoposide in previously untreated extensive-stage small cell lung cancer (SCLC) (23). Seventy-four patients were randomized to receive platinum (cisplatin or carboplatin) with etoposide, in combination with vandetanib, 100 mg daily, or placebo, up to four total cycles (no maintenance therapy allowed). Also, this trial was negative, with a median time to progression (TTP) of 5.62 months in the vandetanib arm versus 5.68 months in the placebo arm (p = 0.9518). Median overall survival was 13.24 versus 9.23 months with vandetanib and placebo, respectively (p=0.45). Moreover, the addition of vandetanib increased non hematological toxicity.

### Sunitinib

Sunitinib is a small-molecule tyrosine kinase inhibitor that inhibits VEGF receptors (VEGFRs), platelet-derived growth factor receptor (PDGFR), Flt-3, and Kit (24). This drug was first evaluated in an open label, single-arm phase II trial in ES-SCLC patients progressed during or after at least a platinum based chemotherapy (25). Patients received a starting dose of sunitinib of 50 mg orally once daily in 6-week cycles composed of a 4-week treatment period followed by 2 weeks off treatment (schedule 4/2). Among the 25 enrolled patients, only two reported a PR (ORR 9%); median PFS was 1.4 months (95% CI, 1.1–1.7) and median overall survival was 5.6 months (95% CI, 3.5–7.7), respectively. Moreover, 75% of patients presented grade 3 or 4 toxicities. Therefore, sunitinib did not appear to warrant further evaluation in the second-line setting. Sunitinib was then evaluated in a single-institution phase II trial as maintenance treatment at 50 mg daily in 16 patients after response to platinum-based chemotherapy (26). Median PFS from the start of sunitinib was 2.5 months (95% CI, 0.8–3.1) and the trial was stopped for futility. Moreover, the drug was discontinued in half of patients due to toxicity or request to stop therapy. Another phase II study was conducted with sunitinib at a lower dose (25 mg daily) as maintenance treatment in 17 patients with ES-SCLC responding to first-line chemotherapy with irinotecan (60 mg/m2, days 1, 8, 15) and carboplatin (AUC = 4, day 1) for six cycles (27). The results of this trial were encouraging, with a median time to progression of 7.6 months from the beginning of chemotherapy and a 1-year OS of 54%. Moreover, the incidence of grade 3 to 4 toxicities was low with this dose of sunitinib. The results of a randomized phase II study with sunitinib as maintenance therapy has been reported by Ready et al. (28). Patients with ES-SCLC without progression after 4/6 cycles of platinum (cisplatin 80 mg/m2 or carboplatin area under the curve of 5 on day 1) plus etoposide (100 mg/m2 per day on days 1 to every 21 days) were randomly assigned to sunitinib at the dose of 37.5 mg or placebo until progression. A total of 85 of 144 patients were randomized to maintenance treatment. The median progression free survival was improved with sunitinib from 2.1 to 3.7 months after randomization which met the primary end-point for significance (HR, 1.62; 95% CI, 1.02–2.60; one-sided p=0.02). Median OS was not significantly improved by sunitinib (6.9 months for placebo and 9.0 months for sunitinib (HR, 1.28; 95% CI, 0.79–2.10; one-sided p = 0.16). Grade 3/4 toxicity included fatigue (19%), neutropenia (14%), decreased leucocytes (7%), and thrombocytopenia (7%). Finally, another single-arm phase II study evaluated sunitinib as maintenance treatment, with a loading dose of 150 mg followed by 37.5 mg daily, in patients with SCLC who were either chemo naive (ED) or have a sensitive relapse (29). Also, this study was closed early because of low accrual (only 9/48 patients required). Two patients achieved DCR at 8 weeks, but significant toxicity was noted (pulmonary hemorrhage, G3 anorexia, diarrhea, thrombocytopenia).

### Sorafenib

Sorafenib is a multi-target tyrosine kinase inhibitor of Raf kinase, VEGFR, and PDGFR (24). This drug was tested in a phase II trial to evaluate whether the combination of standard chemotherapy with cisplatin and etoposide (EC) plus concurrent and sequential sorafenib could prolong survival in patients with previously untreated SCLC (30). A total of 18 patients were enrolled and 17 were treated with four cycles of EC plus concurrent sorafenib 200 mg orally bid. Those patients without progression continued sorafenib 400 mg orally bid as maintenance for maximum of 12 months. The combination of EC and sorafenib showed a limited activity, with an overall median survival of 7.4 months and 1 year survival of 25% and significant toxicity, with a grade 5 gastrointestinal bleeding, pulmonary hemorrhage, and neutropenia in 1 patient.

### Cediranib

Cediranib is a potent and selective inhibitor of the VEGFR-1, -2, and -3 and c-kit. It was evaluated in a phase II trial in patient with refractory or recurrent SCLC (31). Twenty five patients were enrolled, the first 12 patients were treated with a dose of cediranib of 45 mg PO QD, the subsequent patients was treated with a dose reduction to 30 mg PO QD, due to intolerance of the higher dose. Treatment was given on a daily continuous schedule. Tolerability was better with the lower dose (30 mg/day). However, no objective responses were observed at either of the dose levels of cediranib that failed to demonstrate activity in pretreated SCLC patients.

### Nintedanib

Nintedanib is a potent oral triple angiokinase inhibitor targeting VEGFR1–3, PDGFR α-β, and FGFR 1–3 (32). Given the potential activity through the inhibition of angiogenesis and a favorable toxicity profile, it was evaluated in a phase II study in patients with relapsed or refractory SCLC (33). Patients with ES-SCLC who progressed during or after treatment with at least one platinum-based chemotherapy were enrolled in this single-arm phase II trial and received nintedanib 200 mg orally twice daily every 4 weeks. A total of 24 patients were enrolled and 22 were evaluated for response. The ORR was 5% (95% CI, 0.1–22.8). The median PFS was 1.0 (95% CI, 0.9–1.1) month, and OS was 9.8 (95% CI, 8.4–11.2) months. Nintedanib had a manageable AE profile, but a limited activity in relapsed or refractory SCLC.

### Thalidomide

Thalidomide, a glutamic acid derivative, inhibits angiogenesis by repression of key angiogenic genes and downregulation of VEGFR and basic FGF secretion (34). In addition, preclinical models have demonstrated synergistic activity when thalidomide is combined with cytotoxic agents. Mall et al. reported a clinical case of a long survival in a patient with ES-SCLC, treated with chemotherapy and thalidomide, underlining the possible role of angiogenesis inhibitors in combination with traditional chemotherapy in the SCLC (35). A phase II trial evaluated thalidomide as maintenance therapy in 30 patients with ES-SCLC who had received first-line chemotherapy without progression disease (36). Patients received thalidomide 200 mg daily starting 3 to 6 weeks after completion of chemotherapy. The results showed that thalidomide was well tolerated, with grade 1 neuropathy in 27% of patients and only one case of grade 3 neuropathy. Median survival was 12.8 months (95% CI, 10.1–15.8 months) and 1-year survival 51.7% (95% CI, 32.5–67.9%). Another single-arm phase II study evaluated the activity of the combination of thalidomide and chemotherapy in 25 chemotherapy-naive patients with extensive stage or limited stage SCLC (37). Patients were treated with carboplatin and etoposide every 3 weeks for six cycles with concurrent thalidomide 100 mg orally daily. The treatment with thalidomide was continued as maintenance for up to 2 years. The treatment appeared well tolerated and the results on survival and tumor response rate with an ORR of 68% (95% CI, 46–85%), including four complete remissions (20%) and 13 partial remissions (48%) led to the initiation of a randomized phase III trial (38). This prospective, randomized, double-blind, placebo-controlled phase III study enrolled 119 patients who received two courses of etoposide, cisplatin, cyclophosphamide, and epidoxorubicin (PCDE). Responders (92 patients) were randomized to receive four additional PCDE cycles plus thalidomide (400 mg daily) or placebo. The study did not show a statistically significant difference in survival, although patients treated with thalidomide had a longer survival compared with patients treated with placebo (median OS, 11.7 versus 8.7 months, respectively; HR, 0.74; 95% CI, 0.49–1.12; p = 0.16). Patients with a worst performance status (1 or 2) had a significantly longer overall survival (HR, 0.59; 95% CI, 0.37–0.92; p = 0.02) and a slower progression of disease (HR, 0.54; 95% CI, 0.36–0.87; p = 0.02) when received thalidomide, whereas the difference did not reach statistical significance for the whole population (HR, 0.74; 95% CI, 0.49–1.12; p = 0.15). Neuropathy occurred more frequently in the thalidomide than in the placebo group (33% versus 12%, respectively). The combination of thalidomide plus chemotherapy was then evaluated in a large randomized, double-blind, placebo controlled phase III trial (39). A total of 724 patients were randomly assigned to receive placebo or thalidomide 200 mg daily for up to 2 years with concomitant etoposide and carboplatin every 3 weeks for up to six cycles. Unfortunately, the combination of thalidomide plus chemotherapy did not improve overall survival (10.5 versus 10.1 months for placebo and thalidomide, respectively, HR, 1.09; 95% CI, 0.93–1.27; p = 0.28), but it was associated with an increased risk of thrombotic events, mainly pulmonary embolus and deep vein thrombosis (19% versus 10% in thalidomide versus placebo; HR, 2.13; 95% CI, 1.41–3.20; p < 0.001).

### Anlotinib

Anlotinib is a new orally administered tyrosine kinase inhibitor that targets vascular endothelial growth factor receptor (VEGFR), fibroblast growth factor receptor (FGFR), platelet-derived growth factor receptors (PDGFR) and c-kit, approved by China Food and Drug Administration (CFDA) in 2018 as third-line treatment for advanced NSCLC on the bases of the ALTER 0303 study (40). In this randomized phase III study anlotinib significantly prolonged OS, PFS, and RR in 439 Chinese patients with advanced NSCLC, progressing after second-line or further treatment. A subsequent randomized, double-blind, placebo-controlled, phase II study was conducted in 120 Chinese patients with advanced SCLC as a third-line or beyond treatment (ALTER 1202 study) (41). Patients were randomized in a 2:1 ratio to anlotinib (12 mg/daily, orally, 2 weeks on and 1 week off) or placebo. The study met the primary end-point (PFS): median PFS was 4.3 versus 0.7 months in the anlotinib and placebo group, respectively (HR, 0.19; p<0.0001). Moreover, anlotinib prolonged OS (7.3 versus 4.9 months) and improved DCR (71.6% versus 13.2%). Grade 3 to 4 adverse events were slightly more frequent in anlotinib than placebo group, with hemoptysis being the most serious complication. Consequently, anlotinib was approved as the standard third-line therapy for patients with ES-SCLC by the CFDA in 2019. The activity and safety of anlotinib for patients with ES-SCLC who failed at least two lines of previous systemic therapy was recently confirmed by a retrospective analysis conducted in Chinese patients (42). Overall, 79 elderly patients were evaluable: the ORR was 8.9% and DCR was 69.6%. Median PFS was 3.0 months (95% CI, 2.02–3.98) and median OS was 7.1 months (95% CI, 5.07–9.13). The more frequent adverse events were: hypertension (40.5%), hand-foot syndrome (31.6%), diarrhea (27.8%), anorexia (20.3%), fatigue (17.7%), and weight loss (17.7%). Interestingly, a prolonged PFS was observed in patients developing hypertension (4.35 versus 2.95 months, respectively; p=0.01) or hand-foot syndrome (4.20 versus 2.95 months, respectively; p=0.03).

### Apatinib

Apatinib is a new potent oral inhibitor of VEGFR-2, c-kit, and c-src that has demonstrated activity in Chinese patients with hepatocellular carcinoma, gastric cancer, and also ES-SCLC (43–46). A phase II, multicenter study evaluated the activity of apatinib at 500 mg once daily in 40 patients with ES-SCLC who had progressed after two or three previous therapies. An objective response was observed in 17.5% patients; median PFS and OS were 3.0 and 5.8 months, respectively. The safety profile was acceptable, and no grade 5 AEs were reported (45). Similar findings were reported by another smaller, single center, phase II study, conducted in 22 patients with heavily pretreated ES-SCLC treated with 500 mg apatinib. An objective response was observed in 13.6% patients and a disease control rate in 95.5% patients; median PFS and OS were 5.4 and 10.0 months, respectively. No grade 4 and grade 5 AEs were observed. Hypertension and proteinuria were the most common AEs. Moreover, multivariate analysis showed that secondary hypertension was an independent predictor of OS (p = 0.047) (46). A subsequent multicenter, open-label, phase 2 trial (PASSION) investigated the activity and safety of apatinib plus camrelizumab (a PD-1 inhibitor that has demonstrated a promising activity in patients with hepatocellular carcinoma), in patients with ED-SCLC in progression after first-line platinum based chemotherapy (47). A total of 59 patients were enrolled, with 47 patients in the QD cohort (camrelizumab 200 mg every 2 weeks plus apatinib 375 mg once daily). An objective response was observed in 34% patients; median PFS and OS were 3.6 and 8.4 months, respectively. The toxicity profile was acceptable: the most common severe AEs were hypertension, decreased platelet count and hand-foot syndrome. This was the first phase 2 study evaluating the combination of an immune checkpoint inhibitor with anti-VEGFR in patients with advanced SCLC and the reported positive results support further clinical studies of camrelizumab plus apatinib in SCLC.

## Discussion

**Figure 1:**
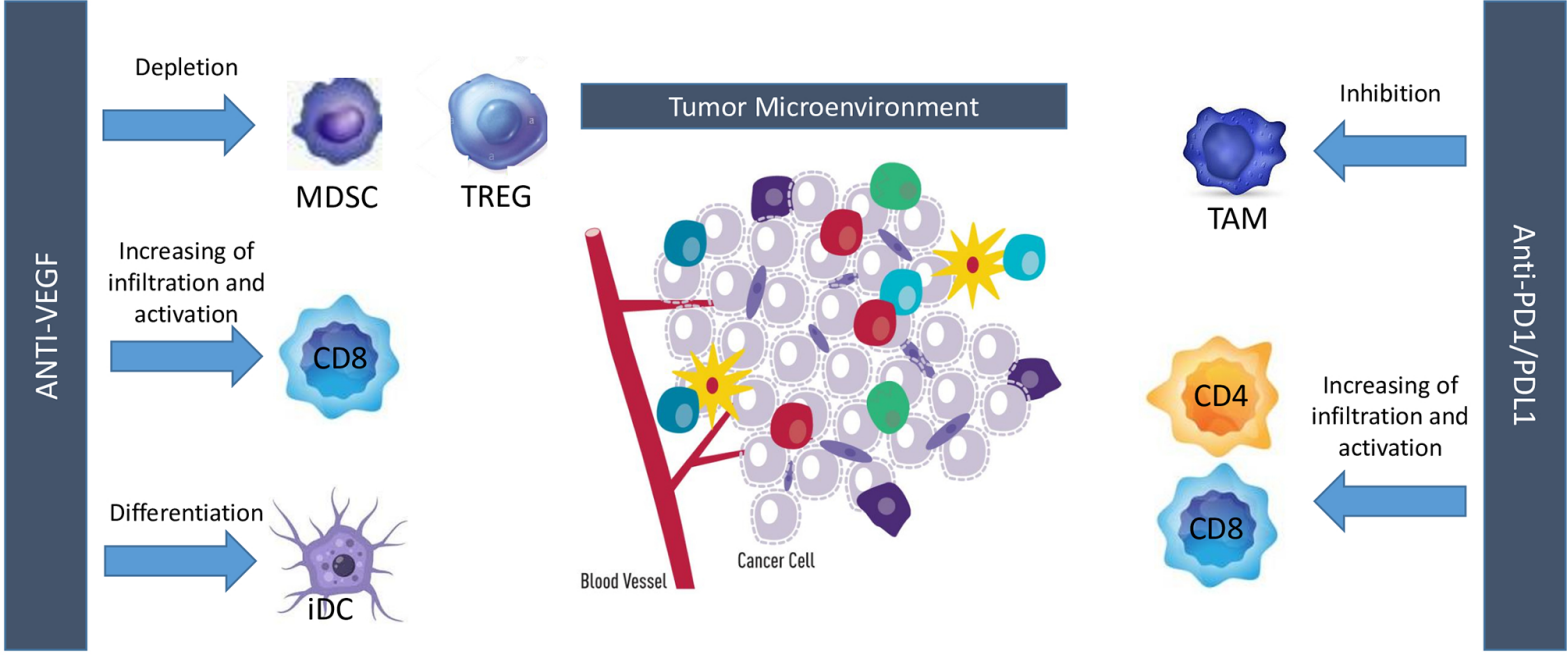
Interactions between angiogenesis and immune-checkpoint inhibitors. MDSC, myeloid-derived suppressor cell; TREG, T regulatory cells; iDC, inhibitory dendritic cells; TAM, tumor-associated macrophages.

Treatment with angiogenesis inhibitors showed contrasting results in patients with SCLC. In patients with LD-SCLC, negative results were observed with bevacizumab as maintenance therapy following treatment with carboplatin, irinotecan, and concurrent radiation. In patients with ES-SCLC, bevacizumab showed limited activity as first-line therapy, in combination with carboplatin/cisplatin and irinotecan (LUN90), or after two cycles of induction chemotherapy (IFCT- 0802 trial). On the contrary, a promising activity was observed in the first-line setting with bevacizumab in combination with etoposide and cisplatin and two randomized studies confirmed that bevacizumab improved PFS, but not OS (SALUTE and GOIRC studies). In particular, in the GOIRC study, a significant overall survival advantage was observed in patients treated with bevacizumab as maintenance treatment, supporting the hypothesis of further testing antiangiogenic agents in the maintenance setting. Instead, disappointing results have been observed with the other antiangiogenic agents, with the exception of anlotinib. In particular, negative results were reported with endostar, sunitinib, sorafenib, vandetanib, or thalidomide in combination with chemotherapy in the first-line setting, with sunitinib as maintenance therapy, with sunitinib, cediranib, and nintedanib as single agents or ziv-aflibercept in combination with topotecan in second-line setting. A meta-analysis of seven randomized controlled trials including 1322 patients concluded that treatment with angiogenesis inhibitors was not associated with improvement of PFS, OS, and ORR, but increased incidence of constipation and embolism in SCLC (48). A subgroup analysis confirmed that bevacizumab improved only PFS (HR, 0.73; 95% CI, 0.42–0.97; p = 0.04). Therefore, existing data do not support currently the use of angiogenesis inhibitors in SCLC, except for anlotinib that improved OS and PFS as third-line therapy in Chinese patients with ES-SCLC and it was approved with this indication in China. However, the scenario of treatment of SCLC is quickly changing in the last few years, due to the positive results observed with the combination of immune checkpoint inhibitors plus chemotherapy in the first-line setting and the recent approval by the Food and Drug Administration of lurbinectedin in patients pretreated with platinum based chemotherapy (3, 49). Is there still a place for angiogenesis inhibitors in this new scenario of treatment of SCLC? Preclinical studies have highlighted a consistent and complex cross-talk between angiogenic molecules and immune cells. For instance, VEGF and HGF directly inhibit dendritic cells maturation and hematopoietic cell differentiation into CD8+ and CD4+ T cells. Moreover, this molecule also enhances both PD-L1/PD-1 expression and immunosuppressant cells infiltration in tumor microenvironment, notably represented by Treg cells and myeloid-derived suppressor cells (MDSCs). VEGF also exerts a key role in modulating the expression of some adhesion molecules on endothelial cells, thus limiting diapedesis of certain immune cells into tumor bed (50). Taken together, these data support the hypothesis of a direct inhibitory effect exerted by angiogenic molecules on immune system. On the other hand, evidences suggest that immuno-related pro-inflammatory cytokines, such as IL-1, IL-6, and IL-17, and tumor-derived chemokines may determine an augmented VEGF production with the aforementioned results. Overall, these data highlight a bi-directional crosstalk between angiogenic molecules and immune system which eventually translates into tumor escape from immune-surveillance (51). Since both anti-angiogenic drugs and immuno-checkpoint inhibitors eventually results in a lower immune-suppressive tumor microenvironment and taking into account the aforementioned crosstalk, combined treatment with these two strategies was performed, resulting in a synergistic antitumor activity with major changes in tumor bed. In melanoma patients, adding bevacizumab to ipilimumab determined an augmented rate of CD8+ T cells and CD163+ dendritic macrophages, both on tumor tissue and in peripheral blood samples. Interestingly, patients receiving ipilimumab plus bevacizumab experienced also a major clinical benefit with a significant efficacy of the combined treatment (52). Similar results were also obtained in murine lung adenocarcinoma models treated with anti-VEGFR2 (apatininb) and anti-PD-L1. Compared with apatinib monotherapy, those treated with combination strategy displayed a lower tumor growth, mainly *via* higher CD8+ T cells infiltration, reduced Treg and MDSC infiltration and lower PD-1/PD-L1 expression (53). To resume, anti-angiogenic drugs increase immune-cell infiltration by inducing vessel normalization and can enable more effector cells, such as CD8+ T cells or natural killer cells, to become activated upon tumor-cell recognition, while immunotherapies can activate effector immune cells that can also secrete pro-angiogenic factors, promoting vascular remodeling (**Figure 1**). This strong biological rationale eventually translated into major clinical benefits so far that this synergistic approach ultimately gained the regulatory approval for several tumor histotypes (54–57).

In mouse models of SCLC, combined treatment with anti-VEGF and anti-PD-L1 agents significantly improved both PFS and OS compared with anti-PD-L1 alone, anti-VEGF alone or with standard combined cisplatin/etoposide chemotherapy. Moreover, acquired resistance to immunotherapy was associated with the PD-1/TIM-3 double-positive exhausted T-cell phenotype in tumor bed and this phenomenon was further abrogated by adding anti-VEGF targeted agents, thus highlighting a potential role of anti-VEGF agents in reverting secondary resistance to immunotherapy. Interestingly, PD-1/TIM-3 double-positive exhausted T-cell phenotype was also found in blood samples of SCLC patients after progression to nivolumab (58). Since VEGFR and PD-L1 are highly found in SCLC patients and taking into account the aforementioned preclinical evidence, several ongoing clinical trials are currently evaluating the efficacy of anti-angiogenic plus immunotherapy agents in SCLC patients (**Table 3**) (59). The phase II PASSION trial was one of the first studies demonstrating the feasibility of this strategy in SCLC and the promising clinical results observed in this trial support the conduction of further clinical studies with angiogenesis and immune checkpoint inhibitors in SCLC (47). A single-arm, prospective study is currently ongoing with apatinib in combination with camrelizumab, etoposide and cisplatin in first-line treatment of ES-SCLC (ClinicalTrial.gov: NCT04490421). Primary outcome is 1 year OS; the planned accrual is 45 patients. Estimated study completion date is February 2022. A phase II-III trial is currently evaluating the activity of anlotinib plus sintilimab (a PD-1 inhibitor which has already been tested with positive results in refractory classical Hodgkin lymphoma) in second or above lines in Chinese SCLC patients (ClinicalTrial.gov: NCT04192682). Primary endpoint is PFS, while secondary end points are OS and ORR. Estimated enrollment is 40 patients. Results are awaited for July 2021. The ETER701 is a phase 3 trial evaluating the efficacy of anlotinib plus carboplatin and etoposide plus placebo or TQB2450 (an anti-PDL1 antibody) versus carboplatin or etoposide (ClinicaTrial.gov: NCT04234607). The trial will enroll 738 naive extended SCLC patients. Anlotinib plus TQB2450 or placebo will be administered as maintenance therapy after the end of chemotherapy treatment. Primary end points are PFS and OS. Results are awaited for January 2023. A phase 2 trial is evaluating vorolanib, a potent oral tyrosine kinase inhibitor which is active on VEGFR and PDGFR, as maintenance in association with atezolizumab in patients with ES-SCLC with no evidence of progression after 3 or 4 cycles of standard of care therapy with carboplatin plus etoposide plus atezolizumab (ClinicalTrial.gov: NCT04373369). Primary outcome measure is PFS. Other trials are ongoing with new angiogenesis inhibitors in combination with standard chemotherapy in patients with SCLC.

**Table 3:** Ongoing trials with angiogenesis inhibitors in ES-SCLC.

Trial	Phase	Setting	Pts	Treatment	Primary end points
**NCT04192682**	Phase II/III	Second or subsequent line	40	Anlotinib + sintilimab	PFS
**NCT04234607**	Phase III	First line	738	Anlotinib + Carboplatin + Etoposide + TQB2450 vs Anlotinib + Carboplatin + Etoposide + Placebo vs Carboplatin + Etoposide	PFS; OS
**NCT04073550**	Phase III	Second line	184	Anlotinib + topotecan vs placebo + topotecan	PFS
**NCT02875457**	Phase III	First line	100	Apatinib + etoposide + cisplatin vs placebo + etoposide + cisplatin	PFS
**NCT04490421**	Phase III	First line	45	Apatinib + etoposide + cisplatin + camrelizumab	1 year OS
**NCT04254471**	Phase II/III	First line	313	Lucitanib + carboplatin + etoposide vs Placebo + Carboplatin + Etoposide	AE (phase 2); PFS (phase 3)
**NCT04373369**	Phase II	First line, maintenance	33	Vorolanib + atezolizumab after 3 or 4 cycles of carboplatin + etoposide + atezolizumab	PFS

A number of clinical trials are also evaluating the efficacy of anti-angiogenic agents in combination with chemotherapy in SCLC. A phase 3 trial is currently evaluating the efficacy of anlotinib plus topotecan versus placebo plus topotecan in SCLC patients who have experienced a progression after platinum treatment (ClinicalTrial.gov: NCT04073550). Primary end point is PFS; 184 patients will be enrolled. Estimated end of trial is for August 2022. A phase 3 trial is evaluating the efficacy of apatinib in combination with etoposide and cisplatin versus placebo plus etoposide and cisplatin in ES-SCLC patients (ClinicalTrial.gov: NCT02875457). Primary outcome is PFS; the trial estimated enrollment is of 100 patients. Results are awaited for October 2023. Lucitanib, another oral multi-tyrosine kinase inhibitor, which targets VEGR, FGFR, and PDGFR, is currently under investigation in a phase II-III trial in combination with carboplatin plus etoposide versus carboplatin and etoposide plus placebo (ClinicalTrial.gov: NCT04254471). Primary outcome in the phase II part of the trial are adverse events; in the phase III part of the trial, primary outcome is PFS. The estimated enrollment is 313 patients. Results are awaited for 2023.

Another relevant issue is the lack of predictive molecular biomarkers to date for angiogenesis inhibitors. In 2015, Zhang et al. demonstrated that lower serum levels of angiopoietin-2 in SCLC patients correlated with better survival benefit and better response to chemotherapy (60). Even though VEGF-expressing cells and serum VEGF/VEGFR levels have been associated with worse survival outcomes in some small studies performed in SCLC patients (61, 62), these molecules failed to be identified as predictive biomarkers for bevacizumab in the IFCT-0802 trial (15). Moreover, no correlation was seen between vascular endothelial growth factor genotypes and outcomes with vandetanib in combination with chemotherapy in the LUN06- 113 study (23). Finally, to date no predictive factors in response to immunotherapy for SCLC patients have been identified. In IMpower 133 trial, both PD-L1 expression and TMB status poorly correlated with clinical outcomes of SCLC patients treated with atezolizumab plus chemotherapy, making the field of predictive biomarkers for combined therapies even more challenging (63).

## Conclusions

Angiogenesis inhibitors have demonstrated to date a limited benefit in SCLC: bevacizumab improved PFS, but not OS when combined to first-line chemotherapy and anlotinib prolonged OS and PFS as third-line therapy in Chinese patients with ES-SCLC. Ongoing trials with checkpoint inhibitors in combination with chemotherapy and angiogenesis inhibitors should definitively define the role of these agents in the first-line setting of treatment of ES-SCLC. The identification of predictive biomarkers of response to angiogenesis and immune-checkpoint inhibitors is an important goal of future research to optimize the use of these agents in SCLC.

## Author Contributions

Conceptualization, AMon and AMor. Writing—original draft preparation, AMan, GC, GP, GE, AMor, and AMon. Writing—review and editing, AMan, GC, GP, GE, VS, RC, CS, GB, MP, PC, GPasc, NN, AMor, and AMon. Supervision, AMor. All authors contributed to the article and approved the submitted version.

## Conflict of Interest

AMor declares the following conflicts of interest: Speaker’s fee: MSD, BMS, Boehringer, Pfizer, Roche, AstraZeneca; Advisory Board: Takeda. NN declares the following personal financial interests (speaker’s fee and/or advisory boards): MSD, Qiagen, Bayer, Biocartis, Incyte, Roche, BMS, MERCK, Thermofisher, Boehringer Ingelheim, Astrazeneca, Sanofi, Eli Lilly; Institutional financial interests (financial support to research projects): MERCK, Sysmex, Thermofisher, QIAGEN, Roche, Astrazeneca, Biocartis. Non-financial interests: President, International Quality Network for Pathology (IQN Path); President, Italian Cancer Society (SIC).

The remaining authors declare that the research was conducted in the absence of any commercial or financial relationships that could be construed as a potential conflict of interest.
